# Remote data collection speech analysis in people at risk for Alzheimer's disease dementia: usability and acceptability results

**DOI:** 10.3389/frdem.2023.1271156

**Published:** 2023-10-13

**Authors:** Sarah Gregory, John Harrison, Janna Herrmann, Matthew Hunter, Natalie Jenkins, Alexandra König, Nicklas Linz, Saturnino Luz, Elisa Mallick, Hannah Pullen, Miles Welstead, Stephen Ruhmel, Johannes Tröger, Craig W. Ritchie

**Affiliations:** ^1^Edinburgh Dementia Prevention, Centre for Clinical Brain Sciences, University of Edinburgh, Edinburgh, United Kingdom; ^2^Scottish Brain Sciences, Edinburgh, United Kingdom; ^3^Department of Neurology, Alzheimer Center Amsterdam, Amsterdam University Medical Centers, Vrije Universiteit, Amsterdam, Netherlands; ^4^King's College London, Institute of Psychiatry, Psychology and Neuroscience, London, United Kingdom; ^5^ki:elements GmbH, Saarbrücken, Germany; ^6^Institute of Neuroscience and Psychology, University of Glasgow, Glasgow, United Kingdom; ^7^CoBTek (Cognition-Behaviour-Technology) Lab, Université Côte d'Azur, Nice, France; ^8^Usher Institute, University of Edinburgh, Edinburgh, United Kingdom; ^9^Janssen Research & Development, LLC, Raritan, NJ, United States

**Keywords:** speech, biomarkers, Alzheimer's disease, cognition, acceptability, feasibility

## Abstract

**Introduction:**

Digital cognitive assessments are gathering importance for the decentralized remote clinical trials of the future. Before including such assessments in clinical trials, they must be tested to confirm feasibility and acceptability with the intended participant group. This study presents usability and acceptability data from the Speech on the Phone Assessment (SPeAk) study.

**Methods:**

Participants (*N* = 68, mean age 70.43 years, 52.9% male) provided demographic data and completed baseline and 3-month follow-up phone based assessments. The baseline visit was administered by a trained researcher and included a spontaneous speech assessment and a brief cognitive battery (immediate and delayed recall, digit span, and verbal fluency). The follow-up visit repeated the cognitive battery which was administered by an automatic phone bot. Participants were randomized to receive their cognitive test results acer the final or acer each study visit. Participants completed acceptability questionnaires electronically acer each study visit.

**Results:**

There was excellent retention (98.5%), few technical issues (*n* = 5), and good interrater reliability. Participants rated the assessment as acceptable, confirming the ease of use of the technology and their comfort in completing cognitive tasks on the phone. Participants generally reported feeling happy to receive the results of their cognitive tests, and this disclosure did not cause participants to feel worried.

**Discussion:**

The results from this usability and acceptability analysis suggest that completing this brief battery of cognitive tests via a telephone call is both acceptable and feasible in a midlife-to-older adult population in the United Kingdom, living at risk for Alzheimer's disease.

## 1. Introduction

With neurodegenerative disease continuing to grow as a major global public health concern (Nichols et al., 2019), identifying risk factors and early biomarkers of neurodegenerative diseases has become increasingly important (Livingston et al., 2017, 2020). Currently, biomarkers for Alzheimer's disease (AD), the most common cause of dementia, tend to be expensive and invasive for the patient or research participant (Peskind et al., 2005; Wittenberg et al., 2019). Commonly used AD biomarkers include amyloid positron emission tomography scans (Chételat et al., 2020), cerebrospinal fluid amyloid and tau levels (Blennow and Zetterberg, 2018; Blennow et al., 2019), and plasma amyloid and tau concentrations (Hampel et al., 2018). These biomarkers are largely limited to specialist hospitals and are not globally accessible. Investigating speech as an AD biomarker may be an important contribution to decentralizing screening and profiling tools on a global scale.

Speech data are relatively easy to collect and require technological solutions that do not rely on specialist staff and facilities. Furthermore, it is a non-invasive and safe technique that may be more acceptable to patients, participants, and the public compared to currently used AD biomarkers, as a first procedure to identify those most likely to be AD biomarker positive. Previous studies have found important associations between features of speech and AD biomarkers (Boschi et al., 2017). Speech markers of interest include semantic processing errors, failure-to-stop errors, changes in connected speech, and declines in syntactic complexity (Ahmed et al., 2013; Venneri et al., 2018; Gollan et al., 2020; Mueller et al., 2021). Assessments of cognition made using speech and language technology have been found to be at least as equally discriminative between different groups when compared to traditional neuropsychological assessment (Garcia et al., 2020). These studies have collected data through face-to-face interactions between researchers and participants, which may again perpetuate the barriers to traditional biomarker testing access. Harnessing technology to gather these speech data remotely, be that via the telephone or videoconferencing platforms, will be an important step in widening access to research opportunities. This may even include digital assistants, such as Alexa, Siri, and Google Assistant, which collect “ambient intelligence” but would need to be considered within a well-designed ethical framework (Simon et al., 2022). These benefits are particularly relevant with the move to decentralized trials and trial access to traditionally underserved groups.

Evidence from studies collecting data remotely has shown comparative performance to human evaluators when making cognitive screening decisions (König et al., 2018; Konig et al., 2018; Themistocleous et al., 2020). A recent review by our group found that remote administration of cognitive tests (via telephone or video-calling) was typically consistent with in-person administration but variable and limited at the person/test level, with stronger evidence existing for videoconferencing over telephone calls, highlighting a need for further research in this area (Hunter et al., 2021). The COVID-19 pandemic drove the need to further explore the feasibility of conducting cognitive assessments via remote means, again with videoconferencing methods more typically considered for the adaptation of traditional cognitive assessments (Geddes et al., 2020). A number of cognitive assessments have also been adapted or even designed to be administered via telephone call with this form of assessment appearing to be viable, although not meant to replace gold-standard in-person evaluations (Carlew et al., 2020).

When developing new digital biomarkers, it should be of high priority that acceptability to users is also evaluated. Successful engagement with a novel tool can only arise when it has a favorable acceptability profile. A previous study using a different tool found that collecting speech data on the phone was acceptable to a group of older adults (Diaz-Asper et al., 2021). This study used a short cognitive task of language fluency, as well as a more narrative speech task, and is a helpful indicator of the likely acceptability of collecting speech biomarkers on the phone. Automated testing in the form of social robot administration and smartphone applications have previously been reported to be most feasible and acceptable to participants (Takaeda et al., 2019; Taylor et al., 2023), although in-person human cognitive testing remains mainstream in most clinical and research settings. The recent paradigm shift to digital neuropsychology warrants consideration from validity and ethical perspectives. Barriers to the digitalization of cognitive testing typically focus on operational issues, such as hardware and software barriers, as well as on the validity of the same test across different devices (Germine et al., 2019), with less attention paid to the acceptability of the digital version to patients and research participants. The use of artificial intelligence and machine learning in dementia detection is also a topic of ethical interest. As models derived from these techniques need to be trained on large data sets, these are commonly initially derived in data sets from research participants who often do not represent the general population living with dementia and, as such, have limits to their generalizability (Ford et al., 2023). Included acceptability in the earliest stages of digital biomarker design may be one opportunity to more holistically consider all ethical aspects of these new tools.

Disclosure of participant-level data in research studies is an area of increasing interest, and the routine disclosure of data collected on the phone is an important part of feasibility and acceptability testing. Previous studies suggest that there is a public demand for greater information about their risk factors and current disease status regarding AD, particularly surrounding disclosure of *APOE*ε4 genotype and AD biomarkers (Unterman et al., 1993; Caselli et al., 2014; Ott et al., 2016). A number of studies have investigated the psychological impact of disclosure of genotype and biomarkers and typically found no associations with long-term psychological distress or risks of anxiety or depression (Green et al., 2009; Lim et al., 2016; Burns et al., 2017; Taswell et al., 2017; Wake et al., 2017; Grill et al., 2020). However, some studies have identified short-term adverse outcomes alongside positive experiences (Vanderschaeghe et al., 2017; Largent et al., 2020). Thus, there is a need to provide psychological safety considerations to prevent possible harm. Cognitive test results completed in research settings are rarely fed back, particularly not to healthy participants or those with mild cognitive impairment (MCI). Nevertheless, cognitive tasks are commonly utilized, and anecdotally many participants express interest in their personal test performance. Understanding the acceptability of providing these test results and the consequences is an important consideration.

The Speech on the Phone Assessment (SPeAk) study (Gregory et al., 2022) was designed to collect speech data on the phone at two time points from participants at risk for AD, using both semi-structured and structured speech tasks. This study aimed to assess the usability and acceptability of the study protocol in this population. There was also an analysis of the acceptability of receiving cognitive test results in the context of a research setting.

## 2. Methods

### 2.1. Study design

The SPeAk study was a prospective observational study. Fully described in a protocol paper (Gregory et al., 2022), briefly, the study involved participants completing a baseline and 3-month follow-up visit. An iPad with the Mili platform (previously called the Delta Testing App) was used to place the phone calls to participants and facilitate the assessments. The Mili platform has been validated in a previous study for the semantic fluency task (Tröger et al., 2018, 2019) and the speech biomarker for cognition (Tröger et al., 2022). The first visit was completed with a trained research assistant delivering verbatim task instructions, while the second visit used an automated voice to provide the instructions. Audio of both visits was recorded in the app.

### 2.2. Ethics

The SPeAk study was reviewed and given a favorable ethical opinion by the Edinburgh Medical School Research Ethics Committee (REC Reference 20-EMREC-007).

### 2.3. Participants

Participants were eligible for inclusion in the study if they had previously engaged in cognitive testing at the research site, through either the European Prevention of Alzheimer's Dementia Longitudinal Cohort Study (EPAD LCS; Ritchie et al., 2016, 2020) or the CHARIOT-Pro Substudy (Udeh-Momoh et al., 2021). Participants in these studies represent a spectrum of risk for future development of AD dementia from healthy volunteers to those with MCI. Based on previous study eligibility criteria, participants were aged 50 years or older, fluent in English, and did not have a diagnosis of dementia (although could have a diagnosis of MCI). For the SPeAk study, participants were required to have the capacity to provide informed consent, have access to a phone line (either a mobile phone or a landline), and be available to participate in both visits. As participants were required to have sufficient hearing ability to participate in cognitive testing for previous studies, there were no specific eligibility criteria related to hearing in this protocol. The primary aim of the study was to evaluate an algorithm predicting amyloid and tau status using speech features. The sample size calculation suggested a minimum of 66 participants was required to provide sufficient power for the primary aim. The results reported in this article reflect secondary outcome measures, and as such, there is the potential for any findings to be underpowered, as the overall sample size calculation was not conducted for these outcomes.

### 2.4. Recruitment

Study information was emailed to participants previously enrolled in the EPAD or CHARIOT-Pro Substudy who had provided consent to be contacted about future study opportunities. Participants were provided with an opportunity to ask questions about the study, either via email or a follow-up telephone call. Participants provided electronic consent using Online Survey software (https://www.onlinesurveys.ac.uk/), with consent verbally re-affirmed by the research assistant at the start of the first visit.

### 2.5. Demographics

At the start of the baseline visit, participants verbally reported gender, age, years of education, and current medications to the research assistant.

### 2.6. Cognitive testing

Participants completed cognitive assessments over the phone at the baseline visit and again at 3-month follow-up. At the baseline visit, participants completed two spontaneous speech tasks to provide an opportunity for the collection of more natural speech patterns. The cognitive assessments included three main tasks: list learning (immediate and delayed recall from the Rey Auditory Verbal Learning Test [RAVLT]; Bean, 2011) as well as forward digit span and fluency (phonemic and semantic). During the immediate recall list learning, task participants were read a list of 15 words, 1 per second, and were asked to repeat back as many words as they could remember, with the task repeated five times. After an approximately 10-min delay (during which the digit span and fluency tasks were administered), the participants were asked to recall as many of the 15 words as they could. The digit span task involved participants being read a series of numbers of increasing length (starting from two digits and working up to nine digits) and asked to repeat the series. Each digit sequence length had two trials. Participants completed two fluency tasks, for the semantic fluency task participants were asked to name as many animals as they could think of within 1 min, while for the phonemic fluency task participants were asked to name as many words beginning with the letter *S* as they could within a minute. During the baseline visits, three raters conducted the testing. All underwent the same training and delivered the task instructions verbatim to participants. The second visit was conducted using an automatic phonebot. The first testing session lasted up to 30 min, and the second testing session took ~15 min. All cognitive tasks were selected to represent domains sensitive to decline in early AD.

Speech data were also collected throughout the assessment, although no speech data are included in this current analysis. The speech data were collected by recording the cognitive assessments, from the spontaneous speech task onward. Language and speech variables were extracted on a linguistic and acoustic-level basis, including temporal, voice source, formant, semantic, and synthetic variables.

### 2.7. Results feedback

Participants received feedback after either each testing session or after their study completion and were equally randomized to each feedback condition using a random-number generator prior to enrolment. The feedback included scores on the immediate and delayed recall, digit span, and fluency tasks, with normative scores where available. A brief explanation of the experimental nature of the tasks and support-line phone numbers were included in the results feedback proforma. Any participants who performed outside of expected ranges had their data reviewed by the principal investigator (CWR), and additional feedback phone calls were provided if deemed clinically necessary. Feedback was only provided on the first variable extracted from the speech files (the classical neuropsychological outcome variables) and not on either of the speech features regarding language and speech descriptors.

### 2.8. Acceptability questionnaires

Participants were emailed a copy of an acceptability questionnaire on completion of the baseline and again for the 3-month follow-up visit. These questionnaires (12 questions, 11 Likert-scale questions, and 1 open-ended question or any other comments) asked participants to rate their experience of setting up the study visits, comfort in completing the tests in their home environment, preferences on the cognitive testing format, and experience on receiving results. To encourage participants to feel able to report their honest experiences of study participation, no participant identification was linked to the acceptability questionnaires. See the Supplementary material for acceptability questionnaires.

### 2.9. Data analysis

Descriptive statistics were used to describe the demographic details of the included participants, as well as the floor and ceiling effects of the tests. The test–retest reliability was compared using paired *t*-tests after tests of normality confirmed a normal distribution for all tasks. Change scores were calculated for each task using the Reliable Change Index (RCI). Participants with missing data were pairwise excluded from the final analysis. Summary statistics were used to describe the acceptability questionnaire data, as well as narrative synthesis of qualitative data from free-text fields. Baseline and 3-month responses were compared using paired *t*-tests. The statistical analysis was carried out using Excel and R Studio (Version 2022.07.1+554).

## 3. Results

### 3.1. Demographics

In total, 69 participants consented to participate in the SPeAk study. One participant declined to participate in any protocol activities post-consent, including the provision of demographic data, after changing their minds about completing the cognitive tasks. Thus, the data presented in this article are from the remaining 68 participants. There was a nearly equal split of men and women enrolled in the study (36 men [52.9%] vs. 33 women [47.1%]), with a mean age of 70.43 (*SD*: 6.94) years, and a high average education (16.10, *SD*: 3.68) years. Most participants were living as a couple (*n* = 50, 73.5%). On average, participants were taking 2.09 (*SD* 2.23) medications. Rater 1 completed the majority of the cognitive testing at the baseline visit (*n* = 55, 80.9%), with raters 2 and 3 completing nearly equivalent visits (*n* = 7, 10.3%; *n* = 6, 8.8%, respectively; see Table 1 for full demographic details).

**Table 1:** Descriptive statistics for participants included in the SPeAk study (*N* = 68).

**Variable**	***n* (%)**
Gender: men	36 (52.9)
**Living status**	
Living as a couple	50 (73.5)
Living alone	15 (22.1)
Living with family	3 (4.4)
**Rater**	
1	55 (80.9)
2	7 (10.3)
3	6 (8.8)
**Variable**	**Mean (** ***SD*** **)**
Age (years)	70.43 (6.94)
	Range: 55–88
Education (years)	16.10 (3.68)
	Range: 10–25
Number of medications	2.09 (2.23)
	Range: 0–11

### 3.2. Retention

One participant withdrew from the study between the first and second visit due to not enjoying completing tasks on the phone, resulting in a retention rate of 98.5% (67 of 68 participants completed both visits).

### 3.3. Technological issues

A small number of technical or operational issues were reported during the study, with five technical issues resulting in the follow-up visit completed outside of the protocol window, one network issue causing a failed automated call that was resolved at a later attempt, one participant with hearing difficulties that impaired list learning completion at baseline but not follow-up, and two participants who misheard the letter during the phonemic fluency task during the follow-up visit. This resulted in nine technical or operational issues reported across the 135 visits completed (6.7%).

### 3.4. Cognitive test scores

Other than the withdrawn participant and the participant with hearing impairment at baseline, there were no missing data for any of the cognitive test outcomes. There was a significant decline in performance on the digit span task between baseline and follow-up visit as well as a significant improvement in performance on the phonemic fluency task at the second visit. It is possible that there was an actual decline in digit span abilities in the participant group during the 3-month study period; however, this may also reflect increased difficulty in completing the task when delivered by an automatic phonebot. The increase in performance on the phonemic fluency task is likely to reflect a small learning effect. There were no changes in performance on the immediate or delayed list learning or the semantic fluency tasks. The means, standard deviations, and ranges of test scores are presented in Table 2.

**Table 2:** Table of cognitive test scores at baseline and follow-up visit with comparative statistics included.

**Cognitive test variable**		**Score range**	**Baseline**	**Follow-up**	**Significance tests**
Immediate recall	Mean	0–75	45.27 (13.32)	44.16 (15.29)	*t*: 0.75, *df*: 65, *p*: 0.46
	Range		4–70	2–74	
Digit span	Mean	0–16	10.74 (2.55)	9.34 (2.92)	*t*: 11.78, *df*: 66, *p* < 0.001
	Range		6–16	0–16	
Semantic fluency	Mean	N/A	19.29 (5.64)	19.25 (6.08)	*t*: 0.27, *df*: 66, *p*: 0.79
	Range		2–33	0–31	
Phonemic fluency	Mean		14.53 (4.61)	16.22 (5.07)	*t*: −3.10, *df*: 66, *p*: 0.003^***^
	Range		2–23	2–26	
Delayed recall	Mean	0–15	9.58 (3.40)	9.64 (3.74)	*t*: −0.23, *df*: 65, *p*: 0.82
	Range		0–15	0–15	

^***^p < 0.001.

Analyses were conducted to understand the impact of gender, age, and education (known risk factors for neurodegeneration) on cognitive test performance. Women performed significantly better than men on immediate recall at both baseline and follow-up visits and on delayed memory tasks at the follow-up visit only. At the follow-up visit, women also performed significantly better on the semantic fluency task compared to men. Full details are presented in Table 3. Older age was significantly associated with poorer performance on all tasks except phonemic fluency at both visits and semantic fluency at baseline, where there was no significant association with age. Higher total years of education were associated with higher scores on digit span, phonemic fluency, immediate recall, and semantic fluency. There were no effects of the number of medications on any cognitive test scores.

**Table 3:** Table of cognitive test scores at baseline and follow-up visit by gender.

**Cognitive test variable**		**Baseline**	**β, *SE*, *p***	**Follow-up**	**β, *SE*, *p***
Immediate recall	Men	41.34 (13.97)	−8.22, 3.12, 0.01^*^	38.63 (14.42)	−11.59, 3.48, 0.001^**^
	Women	49.56 (11.28)		50.22 (14.05)	
Digit span	Men	10.89 (2.49)	0.31, 0.62, 0.62	9.20 (2.70)	−0.30, 0.72, 0.68
	Women	10.58 (2.65)		9.50 (3.18)	
Semantic fluency	Men	18.60 (5.68)	−1.43, 1.37, 0.30	17.54 (6.21)	−3.58, 1.43, 0.01^*^
	Women	20.03 (5.58)		21.12 (5.43)	
Phonemic fluency	Men	13.94 (4.98)	−1.21, 1.12, 0.28	15.23 (5.83)	−2.08, 1.22, 0.09
	Women	15.15 (4.17)		17.31 (3.88)	
Delayed recall	Men	8.89 (3.32)	−1.46, 0.82, 0.08	8.43 (3.65)	−2.54, 0.87, 0.005^**^
	Women	10.34 (3.38)		10.97 (3.41)	

^*^p < 0.05; ^**^p < 0.01.

Considering the RCIs for the five cognitive tests, one participant performed worse on all tasks at the follow-up visit. Across most tasks, there was a mix of better, worse, or no change in performance. The exception was for digit span where there was a slight bias toward a worse performance at the follow-up visit. This was particularly noticeable in men compared to women. Those who performed worse on this task were significantly older (71.96 ± 7.13 years) compared to those who performed better (68.36 ± 6.95 years) or had no reliable change (66.00 ± 4.22 years) on the tasks (*p*:0.02). There were no other significant differences in change scores by sex (see Supplementary Table 1), age, or education.

Disclosure status (after both visits or only after the follow-up visit) did not have any significant associations with test scores at the follow-up visit. There was a significant difference between the proportion of participants who performed worse at the follow-up visit for the delayed recall test depending on disclosure status however, as there was no association with the score results (β: 0.01, *SE*: 0.92, *p*: 0.99) this is likely to be a spurious finding.

### 3.5. Interrater reliability

As presented in Table 1, most of the baseline assessments were completed by rater 1 (*n* = 55, 80.9%), with the remaining assessments completed by raters 2 and 3 (*n* = 7, 10.3%, and *n* = 6, 8.8%, respectively). There were no significant differences between participants' cognitive test scores between raters for the memory tasks, digit span, or phonemic fluency. Semantic fluency scores were significantly higher for participants rated by rater 3 compared to rater 1 (β: 6.50. *SE*: 2.73, *p*: 0.02). All data were quality-checked by an independent rater, and no issues were identified, suggesting this is a random effect.

### 3.6. Acceptability

In total, 61 participants completed the acceptability questionnaire after their baseline visit (61/69, 88.4%), and 56 (56/67, 83.6%) completed it following their second visit.

#### 3.6.1. Functionality of software

At both the baseline and follow-up visits, participants found it easy or extremely easy to set up the appointment, and in general, the sound quality of the call was rated as “OK” to “good”. There was a significant difference in the ease of the set-up, with the baseline appointment rated as easier to set up than the follow-up, with no significant differences between ratings of sound between the baseline and follow-up visits. The full results are available in Table 4.

**Table 4:** Functionality of software and comfort with phone appointments.

**Functionality of software and comfort with phone appointments**							
**Question**	**Scale**	**Baseline**			**Follow-up**		
		**Mean**	**Median**	**Range**	**Mean**	**Median**	**Range**
1. How easy or difficult did you find it to set up the phone appointment?	1 extremely difficult, 2 difficult, 3 neither easy or difficult, 4 easy, 5 extremely easy	4.66 (0.68)	5	2–5	4.46 (0.54)	4	3–5
2. How was the sound quality of the phone call?	1 extremely bad, 2 bad, 3 neither good or bad, 4 good, 5 extremely good	3.90 (0.75)	4	2–5	4.11 (0.73)	4	2–5
3. How comfortable did you feel completing the memory and thinking tasks on the phone?	1 extremely uncomfortable, 2 uncomfortable, 3 neither comfortable or uncomfortable, 4 comfortable, 5 extremely comfortable	3.97 (0.75)	4	2–5	3.55 (0.91)	4	1–5
4. How comfortable did you feel completing the memory and thinking tasks within your own home?	1 extremely uncomfortable, 2 uncomfortable, 3 neither comfortable or uncomfortable, 4 comfortable, 5 extremely comfortable	4.36 (0.58)	4	3–5	4.02 (0.82)	4	2–5

Although generally rated as “OK” to “good”, some participants did provide additional feedback about the sound of the calls, both at baseline and follow-up visits, particularly relating to the word list task:

“Better sound quality would be a help—some words were indistinct and there seemed to be a slight echo as if the tester was in an empty room”. *Baseline*

“Sometimes difficult to pick up pronunciation of words over the phone compared to face-to-face”. *Baseline*

“In the computer test if I did not hear a word well I could not retrieve it”. *Follow-Up*

#### 3.6.2. Comfort of participants

Participants were comfortable both with completing the assessments on the phone and completing these tasks within their home environments. Despite scores remaining in the comfortable range at follow-up, significance tests showed that participants reported significantly higher comfort at baseline compared to follow-up both with completing the tasks on the phone and in their own home. The full results are available in Table 4.

Reflecting the generally high rating scores, some participants provided positive feedback in their comments about how it felt to complete the tests at home:

“It was easy to do from home”. *Baseline*

“I did not find it strange doing the questions, conversation via the phone”. *Baseline*

However, other participants provided feedback on feeling less comfortable completing these tests in their own home:

“Although more relaxed I felt less relaxed answering them in my own home which surprised me”. *Baseline*

“At home there are distractions which made me hesitate and [do] less well with some questions”. *Follow-up*

“I was worried someone might knock on my back door or try to test or ring on the phone I was using”. *Follow-up*

#### 3.6.3. Preferences

Despite the ease and comfort of participants in completing these tasks, participants did tend to express a preference for completing these tasks in person—which they had done during previous EPAD or CHARIOT Pro visits compared to on the phone. When asked to consider a preference between a human tester and an automatic phonebot, participants showed a preference for a human tester. These responses suggest that it is the human element that participants enjoy when taking part in research. These results should be considered within the context of the participants at the time of the study being in a government-mandated lockdown due to COVID-19, not social unrest, when social isolation may have been a factor in their preference for seeing or speaking to a real person.

Although there was a clear preference for in-person or with-person testing, participants indicated they would be happy to complete the memory tests on the phone again with either a human or an automatic phonebot. Although participants were generally happy with both settings, when we compared the baseline and follow-up scores, participants were significantly more positive about completing future tests with a human than an automatic phonebot.

When anticipating the second automatic phonebot phone call, participants expected it to be worse or the same as the first call with a human tester, with the actual experience rated as the same as the first call, which is interesting given the preference for the call involving a human tester. A comparison of the baseline and follow-up scores found the follow-up experience was reported as significantly higher than the baseline expectation. The full results are available in Table 5.

**Table 5:** Preferences for human/automatic phonebot tester.

**Preferences for human/automatic phonebot tester**				
**Question**	**Scale**	**Mean**	**Median**	**Range**
5 (Baseline). Thinking back to your previous in-person appointments, did you prefer completing the memory and thinking tasks face-to-face or on the phone?	1 definitely preferred face-to-face, 2 somewhat preferred face-to-face, 3 no preference, 4 somewhat preferred on the phone, 5 definitely preferred on the phone	2.41 (1.05)	2	1–5
5 (Follow-up). Thinking back to your first phone appointment for this study, did you prefer completing the memory and thinking tasks with a person or with a computer?	1 definitely preferred with a person, 2 somewhat preferred with a person, 3 no preference, 4 somewhat preferred with a computer, 5 definitely preferred with a computer	2.09 (1.01)	2	1–5
6 (Baseline). How happy would you be to complete memory and thinking tasks on the phone with a human tester in the future?	1 extremely unhappy, 2 unhappy, 3 neither happy or unhappy, 4 happy, 5 extremely happy	4.28 (0.73)	4	1–5
6 (Follow-up). How happy would you be to complete memory and thinking tasks on the phone with a computer tester in the future?	1 extremely unhappy, 2 unhappy, 3 neither happy or unhappy, 4 happy, 5 extremely happy	3.71 (0.97)	4	2–5
7 (Baseline). Do you expect the second phone appointment with the tests administered by a computer will be better or worse to this first phone appointment?	1 much worse, 2 worse, 3 the same, 4 better, 5 a lot better	2.75 (0.65)	3	1–4
7 (Follow-up). Thinking back to what you expected before this phone appointment, was your experience of a computer tester better or worse than you expected?	1 much worse, 2 worse, 3 the same, 4 better, 5 a lot better	3.20 (0.94)	3	2–5

Comments from participants represented a spectrum of preferences, with some participants commenting on the convenience of phone testing and the ease of completing these tasks having met the tester in person before:

“…the phone appointment is more convenient than traveling for face to face”. *Baseline*

“I think that it was easy to do the tasks over the phone as I have done them face to face before. Moreover, I had met the interviewer/task manager in person in recent years on the... study. I think both of these points had a bearing on my positive attitude to the tasks”. *Baseline*

A majority of participants who provided comments at the follow-up visit showed a clear preference for interacting with a person, either in person or on the phone:

“I prefer the human contact, although the computer was clear and concise”. *Follow-up*

“With the computer, it was hard to gauge how much think-time I had left. With a person, you can see if they are just watching the time.” *Follow-up*

Interestingly, some participants preferred different tasks with different rater types (human and computer):

“Bizarrely, I found the word list easier from the human tester but the number sequences easier from the computer tester.” *Follow-up*

“It is interesting that my computer scores for [list learning] and Digit span are lower than in the human call, because this actually reflects how I felt when doing them. For some reason, the categories were much easier to do with the computer—maybe because I didn't feel so stupid about the words that came into my head.” *Follow-up*

#### 3.6.4. Experience of receiving results from cognitive testing

Participants were interested in receiving their results from the study, regardless of whether they received them after each study appointment or only on study completion. In general, participants reported they did not feel worried about receiving their results and, in fact, tended to report feeling happy to receive them.

Interestingly, participants did not report concerns over the timing of receiving their results, with those who did not receive results after the first session reporting that this did not bother them at all and those who received feedback after each session reporting feeling indifference to this.

Participants were happy with either receiving feedback after each session or at the conclusion, which is helpful for considering the timing of feedback in future studies. It should be noted that any cases in which results fell significantly outside of normative values were discussed with the study's principal investigator, and when required, specific feedback was given regarding recommendations to contact a general practitioner (GP), and referral letters were requested. The full results are available in Table 6.

**Table 6:** Receiving results.

**Receiving results**				
**Question**	**Scale**	**Mean**	**Median**	**Range**
8 (Baseline). If you received your results from the memory and thinking tasks after this phone appointment, how interested were you to receive your study results?	1 not at all, 2 not much, 3 indifferent, 4 a little, 5 a lot, 6 NA	4.46 (0.73)	5	2–5
8 (Follow-up). How interested were you to receive your study results?	1 not at all, 2 not much, 3 indifferent, 4 a little, 5 a lot	4.48 (0.85)	5	2–5
9 (Baseline). If you received your results from the memory and thinking tasks after this phone appointment, did you find it worrying to receive your study results?	1 not at all, 2 not much, 3 indifferent, 4 a little, 5 a lot, 6 NA	2.06 (1.24)	2	1–4
9 (Follow-up). Did you find it worrying to receive your study results?	1 not at all, 2 not much, 3 indifferent, 4 a little, 5 a lot, 6 NA	2.14 (1.21)	2	1–5
10 (Baseline). If you received your results from the memory and thinking tasks after this phone appointment, how happy do you feel about receiving feedback immediately after completing the task?	1 extremely unhappy, 2 unhappy, 3 neither happy or unhappy, 4 happy, 5 extremely happy	3.91 (0.85)	4	1–5
10 (Follow-up). If you received your results only at the conclusion of the study, how happy do you feel about receiving feedback only after completing both phone appointments?	1 extremely unhappy, 2 unhappy, 3 neither happy or unhappy, 4 happy, 5 extremely happy, 6 NA	3.70 (0.70)	4	3–5
11 (Baseline). If you did not receive your results after this phone appointment how much did that bother you?	1 not at all, 2 not much, 3 indifferent, 4 a little, 5 a lot, 6 NA	1.90 (1.03)	2	1–4
11 (Follow-up). If you received your results after both sessions how happy do you feel about receiving feedback after each phone appointment?	1 not at all, 2 not much, 3 indifferent, 4 a little, 5 a lot, 6 NA	3.47 (1.16)	3	1–5

NA, not applicable.

Generally, participants found that receiving results was interesting or reassuring:

“I think you know whether you have completed the tests well, or not, at the time. We all probably think we did worse than is actually true so receiving the results is actually positive as it shows they were not as bad as you thought.” *Baseline*

“I am interested in results but not much bothered and certainly not worried.” *Baseline*

“Getting results was interesting as this did not happen in the face-to-face surveys (at least, not for me).” *Baseline*

However, a few stated that some additional information on how to interpret the results would make these more helpful:

“Very interested to receive the results but would appreciate a bit more explanation—delayed recall—is it better to be higher or lower?” *Follow-up*

“I feel a little explanation in the results I received would help me understand them better, especially the first one; e.g. what do the numbers mean?” *Baseline*

Overall, participants found the process of engaging with a research project involving cognitive tests on the phone easy and comfortable. Although there was a clear preference for in-person, or with-person, testing, participants were happy to repeat on-the-phone testing in the future with either a human or an automatic phonebot. The feedback of results was seen as a positive aspect of the trial and did not appear to cause psychological distress.

## 4. Discussion

The results from this usability and acceptability analysis suggest that completing this brief battery of cognitive tests via a telephone call is both acceptable and feasible in a midlife-to-older adult population in the United Kingdom living at risk for AD. There were no obvious ceiling or floor effects for immediate recall, digit span, or fluency tasks. Some participants did perform at ceiling for the delayed recall task at both baseline and follow-up visits. There was a normal distribution of scores across the cognitive tasks at both visits, suggesting that these tasks perform as we would expect if administered in a face-to-face setting. Previous reviews have found that telephone-based cognitive testing is appropriate in the assessment of cognitive aging (Castanho et al., 2014). Participants enrolled in the study were free of dementia at enrolment. Comparing the scores to studies using the same subtests with in-person administration, we can see participants on average performed in the cognitively normal range (Harrison et al., 2000; Choi et al., 2014; Sirály et al., 2015). A recent review by our group found consistency between in-person and remote administration of cognitive tests for MCI and AD, although noted individual- and test-level variation (Hunter et al., 2021). As this group largely represented a cognitively healthy group, the findings are discussed in this context, with further work needed to understand feasibility and acceptability in populations with more cognitive impairment.

Three of the tasks (immediate recall, delayed recall, and semantic fluency) demonstrated no significant changes between baseline human testing and follow-up automatic phonebot testing, suggesting these are reliable to use in both administration modes. The RCI indicates individual-level changes in performance, which may reflect personal preferences of testing or cognitive fluctuations at the individual level. There were significant differences in performance on the digit span and phonemic fluency tasks between the baseline and follow-up visits. The digit span score significantly decreased between visits. As no other test scores decreased, it is unlikely that the cohort experienced significant cognitive decline in the interim 3-month period, and this decline in score may reflect some hearing, processing, or attentional difficulties when digits were read by the automated voice. This task may need further development before this can be used reliably in a fully automated manner. The increase in the phonemic fluency task may reflect a learning effect, although, interestingly, this was not seen in the semantic fluency task. This improvement in score is driven by women achieving a higher number of words at follow-up compared to baseline, with men's scores decreasing slightly at follow-up. These results reflect data from previous studies, suggesting a small majority of participants improve on a second attempt at fluency tasks (Harrison et al., 2000).

In general, women performed significantly better than men on a number of tasks at baseline and follow-up. This finding aligns with previous literature, where women appear to have an advantage in verbal memory compared to men despite carrying similar pathology burdens (Sundermann et al., 2017). Interestingly, while there is evidence in the literature for women outperforming men on phonemic fluency tasks (Hirnstein et al., 2022), we saw an effect for semantic but not phonemic fluency. It may be that the category of phonemic fluency was advantageous to both men and women to perform well (Hirnstein et al., 2022), leading to a lack of differentiation by gender, and it appears that women had a stronger learning effect compared to men on the semantic fluency task leading to a difference in scores at follow up. Age was significantly associated with most tasks, with education associated with task performance on a small number of tasks with a lack of consistency seen across visits. The importance of gender, age and education should be considered in further work to generate normative scores for these tasks when delivered via the telephone. Education has been reported as associated with performance on these cognitive tasks in previous studies (Zimmermann et al., 2014). Age and gender associations with cognitive test performance for these tasks are inconsistent in previous studies (Van der Elst et al., 2006; Zimmermann et al., 2014; Woods et al., 2016).

Participants found the experience of completing the tasks on the phone to be generally acceptable. The set-up of the appointment was easy for participants to engage with, and participants were comfortable with completing these tasks in their home environment. Although there was an expressed slight preference to complete tasks face-to-face, there was agreement that participants would be happy to repeat these tasks on the phone in the future with either a human or an automatic phonebot tester. Participants appreciated receiving the results of their cognitive tests, and there was no clear preference for receiving these after both visits or at the end of their study participation. While some information was provided on the results proforma, which was co-designed with participant advisors, on what scores might be expected for each task based on in-person testing normative scores, some participants did express a desire for more detailed information. This feedback can inform future studies, giving evidence to support the safe feedback of cognitive test results, with even more detailed proformas to establish the context of what the scores meant.

To the best of our knowledge, this is one of the first studies to evaluate participants' experiences of receiving cognitive test score results. Previous studies have evaluated the effects of disclosing *APOE* genotype and amyloid imaging results to cognitively healthy participants and found there was no increase in psychological distress, anxiety, or depression (Green et al., 2009; Grill et al., 2020). As acceptability of results disclosure was a secondary outcome, we did not gather any data on psychological distress or adjustment before and after results disclosure, and further work should explore this topic in more detail, as well as understand how to present this information to patients and participants in an understandable and meaningful way. However, participants self-reported the experience of receiving the results as overwhelmingly neutral or positive, along with no reports of psychological distress from any participants included in the study, suggesting there is likely little harm caused by disclosing test results from standardized tests used in a different format. As the phonemic fluency task was the only increase in mean scores at the follow-up visit, it is unlikely that result disclosure was associated with any change in test performance. There were a small number of problems that arose due to hearing difficulties. This should be noted as a consideration for wider implementation of phone-based speech and cognitive test data collection.

A key strength of this study was the initial co-design with participant panel members, whose input to the study design and materials was to ensure the project was of interest and comprehensible to the target population. The use of electronic data capture allowed participants to take part in a study during a period when in-person study engagement was limited due to the COVID-19 pandemic. The study has some limitations, with participation restricted to those who had previously engaged in cognitive testing. As such, there is a limit to the generalizability of testing-naïve participants. The participant population was also homogeneous, representing a highly educated cohort, which does not reflect the general population from which they were originally drawn. As this study recruited exclusively from those who had participated in the two earlier studies, there were no mitigations that were able to be put in place for this study; however, future studies should endeavor to explore the use of this technology in diverse populations. As previously mentioned, this should also be tested in a more cognitively impaired population to understand if this form of testing remains feasible and acceptable. It is also important to understand the usability of this tool regarding hearing impairment and the use of hearing aids. Further studies using this tool could include the use of brief hearing tests to establish the level of hearing impairment each participant has as well as record any diagnoses of hearing loss or hearing aid usage. This is important not only as a test that relies on hearing skills but also given the recognized importance of hearing loss as a risk factor for future dementia (Livingston et al., 2020). The testing order was fixed, and as such, we were not able to adjust for test order effects when interpreting changes in test scores.

To conclude, completing a short battery of cognitive tests on the telephone is both feasible and acceptable to an older adult population who are cognitively normal or at risk for future dementia. Future studies are needed to replicate this work in a more diverse participant population with a more pronounced cognitive decline. This study provides initial evidence on the value of feeding back cognitive test results to study participants, with further studies needed to explore more in-depth psychological safety of this approach.

## Data availability statement

The raw data supporting the conclusions of this article will be made available by the authors, without undue reservation.

## Ethics statement

The studies involving humans were approved by Edinburgh Medical School Research Ethics Committee (EMREC). The studies were conducted in accordance with the local legislation and institutional requirements. The participants provided their written informed consent to participate in this study.

## Author contributions

SG: Conceptualization, Data curation, Formal analysis, Methodology, Project administration, Writing—original draft, Writing—review and editing. JHa: Conceptualization, Methodology, Supervision, Writing—review and editing. JHe: Formal analysis, Methodology, Project administration, Writing—review and editing. MH: Data curation, Formal analysis, Methodology, Project administration, Writing—review and editing. NJ: Data curation, Project administration, Writing—review and editing. AK: Conceptualization, Methodology, Writing—review and editing. NL: Conceptualization, Funding acquisition, Methodology, Writing—review and editing. SL: Conceptualization, Methodology, Writing—review and editing. EM: Project administration, Writing—review and editing. HP: Data curation, Methodology, Project administration, Writing—review and editing. MW: Data curation, Project administration, Writing—review and editing. SR: Writing—review and editing. JT: Data curation, Formal analysis, Writing—review and editing. CR: Conceptualization, Funding acquisition, Methodology, Supervision, Writing—review and editing.
