# Supplementary material for: Remote data collection speech analysis in people at risk for Alzheimer's disease dementia: usability and acceptability results
Source: Front Dement. 2023 Oct 13;2:1271156. doi: 10.3389/frdem.2023.1271156 (PMC11285540; doi:10.3389/frdem.2023.1271156)
Supplement: Supplementary file 1 [file Data_Sheet_1.docx]

Supplementary Material

# Supplementary Materials: Acceptability questionnaire: Baseline – (Digitally transcribed into Online Surveys)

A. How easy or difficult did you find it to set up the phone appointment?

1-Extremely difficult

2-Difficult

3-Neither easy or difficult

4-Easy

5-Extremely easy

B. How was the sound quality of the phone call?

1-Extremely bad

2-Bad

3-Neither good or bad

4-Good

5-Extremely good

C. How comfortable did you feel completing the memory and thinking tasks on the phone?

1-Extremely uncomfortable

2-Uncomfortable

3-Neither comfortable or uncomfortable

4-Comfortable

5-Extremely comfortable

D. How comfortable did you feel completing the memory and thinking tasks within your own home?

1-Extremely uncomfortable

2-Uncomfortable

3-Neither comfortable or uncomfortable

4-Comfortable

5-Extremely comfortable

E. Thinking back to your previous in person appointments, did you prefer completing the memory and thinking tasks face to face or on the phone?

1. Definitely preferred face to face

2. Somewhat preferred face to face

3. No preference

4. Somewhat preferred on the phone

5. Definitely preferred on the phone

F. How happy would you be to complete memory and thinking tasks on the phone with a human tester in the future?

1-Extremely unhappy

2-Unhappy

3-Neither happy or unhappy

4-Happy

5-Extremely happy

G. Do you expect the second phone appointment with the tests administered by a computer will be better or worse to this first phone appointment?

1-Much worse

2-Worse

3-The same

4-Better

5-A lot better

H. If you received your results from the memory and thinking tasks after this phone appointment, how interested were you to receive your study results?

1- Not at all

2- Not much

3- Indifferent

4- A little

5- A lot

6. N/A - I did not receive feedback

I. If you received your results from the memory and thinking tasks after this phone phone appointment, did you find it worrying to receive your study results?

1- Not at all

2- Not much

3- Indifferent

4- A little

5- A lot

6. N/A - I did not receive feedback

J. If you received your results from the memory and thinking tasks after this phone appointment, how happy do you feel about receiving feedback immediately after completing the task?

1-Extremely unhappy

2-Unhappy

3-Neither happy or unhappy

4-Happy

5-Extremely happy

6. N/A - I did not receive feedback

K. If you did not receive your results after this phone appointment how much did that bother you?

1- Not at all

2- Not much

3- Indifferent

4- A little

5- A lot

L. Any other comments?

FREE TEXT

# Supplementary Materials: Acceptability questionnaire: Follow up – (Digitally transcribed into Online Surveys)

A. How easy or difficult did you find it to set up the phone appointment?

1-Extremely difficult

2-Difficult

3-Neither easy or difficult

4-Easy

5-Extremely easy

B. How was the sound quality of the phone call?

1-Extremely bad

2-Bad

3-Neither good or bad

4-Good

5-Extremely good

C. How comfortable did you feel completing the memory and thinking tasks on the phone?

1-Extremely uncomfortable

2-Uncomfortable

3-Neither comfortable or uncomfortable

4-Comfortable

5-Extremely comfortable

D. How comfortable did you feel completing the memory and thinking tasks within your own home?

1-Extremely uncomfortable

2-Uncomfortable

3-Neither comfortable or uncomfortable

4-Comfortable

5-Extremely comfortable

E. Thinking back to your first phone appointment for this study, did you prefer completing the memory and thinking tasks with a person or with a computer?

1. Definitely preferred with a person

2. Somewhat preferred with a person

3. No preference

4. Somewhat preferred with a computer

5. Definitely preferred with a computer

F. How happy would you be to complete memory and thinking tasks on the phone with a computer tester in the future?

1-Extremely unhappy

2-Unhappy

3-Neither happy or unhappy

4-Happy

5-Extremely happy

G. Thinking back to what you expected before this phone appointment, was your experience of a computer tester better or worse than you expected?

1-Much worse

2-Worse

3-The same

4-Better

5-A lot better

H. How interested were you to receive your study results?

1- Not at all

2- Not much

3- Indifferent

4- A little

5- A lot

I. Did you find it worrying to receive your study results?

1- Not at all

2- Not much

3- Indifferent

4- A little

5- A lot

J. If you received your results only at the conclusion of the study, how happy do you feel about receiving feedback only after completing both phone appointments?

1-Extremely unhappy

2-Unhappy

3-Neither happy or unhappy

4-Happy

5-Extremely happy

6- N/A - I received feedback after the first phone session

K. If you received your results after both sessions how happy do you feel about receiving feedback after each phone appointment?

1-Extremely unhappy

2-Unhappy

3-Neither happy or unhappy

4-Happy

5-Extremely happy

6- N/A - I received feedback after the second phone session only

L. Any other comments?

FREE TEXT

|  | **Overall cohort (n, %)** | | **By sex (n, %)** | | | **X^2^, p** |
| --- | --- | --- | --- | --- | --- | --- |
| **Immediate Recall** | Better | 24 (36.4) | Men | Better | 10 (28.6) | 2.06, 0.36 |
|  |  |  |  | Worse | 18 (51.4) |  |
|  | Worse | 31 (47.0) |  | No Reliable Change | 7 (20.0) |  |
|  |  |  | Women | Better | 14 (45.2) |  |
|  | No reliable change | 11 (16.7) |  | Worse | 13 (41.9) |  |
|  |  |  |  | No Reliable Change | 4 (12.9) |  |
| **Digit Span** | Better | 11 (16.4) | Men | Better | 7 (20) | 6.24, 0.04* |
|  |  |  |  | Worse | 26 (74.3) |  |
|  | Worse | 45 (67.2) |  | No Reliable Change | 2 (5.7) |  |
|  |  |  | Women | Better | 4 (12.5) |  |
|  | No reliable change | 11 (16.4) |  | Worse | 19 (59.4) |  |
|  |  |  |  | No Reliable Change | 9 (28.1) |  |
| **Semantic fluency** | Better | 26 (38.8) | Men | Better | 14 (40.0) | 2.98, 0.23 |
|  |  |  |  | Worse | 14 (40.0) |  |
|  | Worse | 22 (32.8) |  | No Reliable Change | 7 (20.0) |  |
|  |  |  | Women | Better | 12 (37.5) |  |
|  | No reliable change | 19 (28.4) |  | Worse | 8 (25) |  |
|  |  |  |  | No Reliable Change | 12 (37.5) |  |
| **Phonemic fluency** | Better | 35 (52.5) | Men | Better | 19 (54.3) | 1.25, 0.53 |
|  |  |  |  | Worse | 9 (25.7) |  |
|  | Worse | 15 (22.4) |  | No Reliable Change | 7 (20.0) |  |
|  |  |  | Women | Better | 16 (50.0) |  |
|  | No reliable change | 17 (25.4) |  | Worse | 6 (18.8) |  |
|  |  |  |  | No Reliable Change | 10 (31.2) |  |
| **Delayed Recall** | Better | 32 (48.5) | Men | Better | 13 (37.1) | 4.63, 0.10 |
|  |  |  |  | Worse | 13 (37.1) |  |
|  | Worse | 22 (33.3) |  | No Reliable Change | 9 (25.7) |  |
|  |  |  | Women | Better | 19 (61.3) |  |
|  | No reliable change | 12 (18.2) |  | Worse | 9 (29.0) |  |
|  |  |  |  | No Reliable Change | 3 (9.7) |  |

Supplementary Table 1: Reliable Change Indices of change in performance between baseline and follow up assessment in whole cohort and stratified by sex.
